# PACER: a novel 3D plant cell wall model for the analysis of non-catalytic and enzymatic responses

**DOI:** 10.1186/s13068-022-02128-8

**Published:** 2022-03-16

**Authors:** Mareike Monschein, Edita Jurak, Tanja Paasela, Taru Koitto, Vera Lambauer, Mirko Pavicic, Thomas Enjalbert, Claire Dumon, Emma R. Master

**Affiliations:** 1 Department of Bioproducts and Biosystems, Aalto University, Kemistintie 1, 02150 Espoo, Finland; 2 Department of Bioproduct Engineering, University of Groningen, Nijenborgh 4, 9747 AG Groningen, The Netherlands; 3 Department of Agricultural Sciences, Viikki Plant Science Centre, University of Helsinki, P.O. Box 27, 00014 Helsinki, Finland; 4 Toulouse Biotechnology Institute (TBI), Université de Toulouse, CNRS, INRAE, INSA, 31077 Toulouse, France; 5 Department of Chemical Engineering and Applied Chemistry, University of Toronto, 200 College Street, Toronto, ON M5S 3E5 Canada

**Keywords:** Xylanase, Loosenin, Assay development, Enzyme accessibility, Amorphogenesis, Xylan, Lignocellulose

## Abstract

**Background:**

Substrate accessibility remains a key limitation to the efficient enzymatic deconstruction of lignocellulosic biomass. Limited substrate accessibility is often addressed by increasing enzyme loading, which increases process and product costs. Alternatively, considerable efforts are underway world-wide to identify amorphogenesis-inducing proteins and protein domains that increase the accessibility of carbohydrate-active enzymes to targeted lignocellulose components.

**Results:**

We established a three-dimensional assay, PACER (plant cell wall model for the analysis of non-catalytic and enzymatic responses), that enables analysis of enzyme migration through defined lignocellulose composites. A cellulose/azo-xylan composite was made to demonstrate the PACER concept and then used to test the migration and activity of multiple xylanolytic enzymes. In addition to non-catalytic domains of xylanases, the potential of loosenin-like proteins to boost xylanase migration through cellulose/azo-xylan composites was observed.

**Conclusions:**

The PACER assay is inexpensive and parallelizable, suitable for screening proteins for ability to increase enzyme accessibility to lignocellulose substrates. Using the PACER assay, we visualized the impact of xylan-binding modules and loosenin-like proteins on xylanase mobility and access to targeted substrates. Given the flexibility to use different composite materials, the PACER assay presents a versatile platform to study impacts of lignocellulose components on enzyme access to targeted substrates.

**Supplementary Information:**

The online version contains supplementary material available at 10.1186/s13068-022-02128-8.

## Background

The past 20 years has marked several major breakthroughs in our fundamental understanding of lignocellulose bioconversion [1–3]. Some of these breakthroughs are already incorporated in enzyme formulations for the efficient conversion of lignocellulosic biomass to fuels, chemicals and new bio-based materials [4–6]. Despite the tremendous achievements by excellent research groups world-wide, currently available enzyme formulations fall short of transformation efficiencies achieved in nature. As a result, the economic feasibility of lignocellulose bioconversion to fuels and chemicals remains a challenge [7–9]. In particular, limited accessibility of isolated enzymes to targeted lignocellulose components is acknowledged as a major hurdle to enzymatic deconstruction of biomass [10–14].

Enzyme accessibility to targeted substrates embedded in lignocellulosic fiber (e.g., cellulase accessibility to cellulose) has been correlated to the interior surface area of the fiber (i.e., pore size and pore size distribution) as well as exterior surface area of the fiber (i.e., particle size) [14–18]. In addition to structural factors, the chemical composition and distribution of hemicelluloses and lignin in lignocellulosic substrates impacts enzyme accessibility [19–22]. Measurements of pore size distribution and exterior surface area of lignocellulose substrates include solute exclusion [23, 24], Simons’ staining [10, 25], water retention [26], fiber size analysis [26] and thermoporosimetry [27]. Such methods have been especially beneficial when evaluating the impact of lignocellulose pretreatment and cellulose fiber processing on the performance of cellulolytic enzymes. Besides physicochemical analysis of different lignocellulose preparations, fluorescence microscopy, atomic force microscopy, scanning electron microscopy (SEM) among other imaging techniques are powerful options for monitoring changes to fiber structure and accessibility during enzymatic processing [12, 28–31]. Such imaging approaches, however, are impractical for screening new amorphogenesis-inducing proteins that boost the enzymatic deconstruction of lignocellulose.

The term amorphogenesis was introduced in 1985 to describe the first step to cellulose deconstruction by hydrolytic enzymes [32]. Non-hydrolytic proteins and protein domains that potentially play a role in amorphogenesis increase the initial accessibility of carbohydrate-active enzymes to lignocellulosic substrates [15]. Reported examples include certain carbohydrate-binding modules (CBMs) [33], swollenins [34–36], loosenins [37, 38], microbial expansin-like proteins [38–41] and lytic polysaccharide monooxygenases [42]. Since amorphogenesis-inducing proteins often adopt a non-hydrolytic mode of action, we contend functional screens that rely solely on the detection of soluble products risk overlooking those proteins that impact enzyme accessibility and migration through lignocellulose materials. Accordingly, our aim was to establish a versatile and low-cost approach to screen for proteins that impact the accessibility of glycoside hydrolases to targeted lignocellulose components.

Herein, we introduce the PACER assay, a novel 3D Plant cell wall model for the Analysis of non-Catalytic and Enzymatic Responses. The PACER assay was inspired by a three-dimensional human cell culture system [43] and establishes a versatile new screen for proteins that impact the migration of glycoside hydrolases and other enzymes through model lignocellulosic materials. This technique allows the visualization of enzyme penetration and migration along a constructed cellulose composite, and impacts of non-catalytic proteins and protein modules on enzyme migration. Three lines of investigation were pursued for proof of concept of the PACER assay: (1) comparative analysis of three *endo*-1,4-β-xylanases to assess the impact of protein size and presence of a CBM on enzyme migration through a formulated cellulose/azo-xylan matrix; (2) evaluation of two loosenins for their potential to increase xylanase migration through the formulated matrix, and (3) characterization of *Pm*25, an *endo*-1,4-β-xylanase recently discovered from termite gut metagenome [44]. By simultaneously measuring product release and migration of enzymes through defined cellulosic materials, the PACER assay can uncover determinants of enzyme accessibility not detected through measuring release of soluble products alone.

## Results and discussion

### Development of the PACER assay

**Fig. 1:**
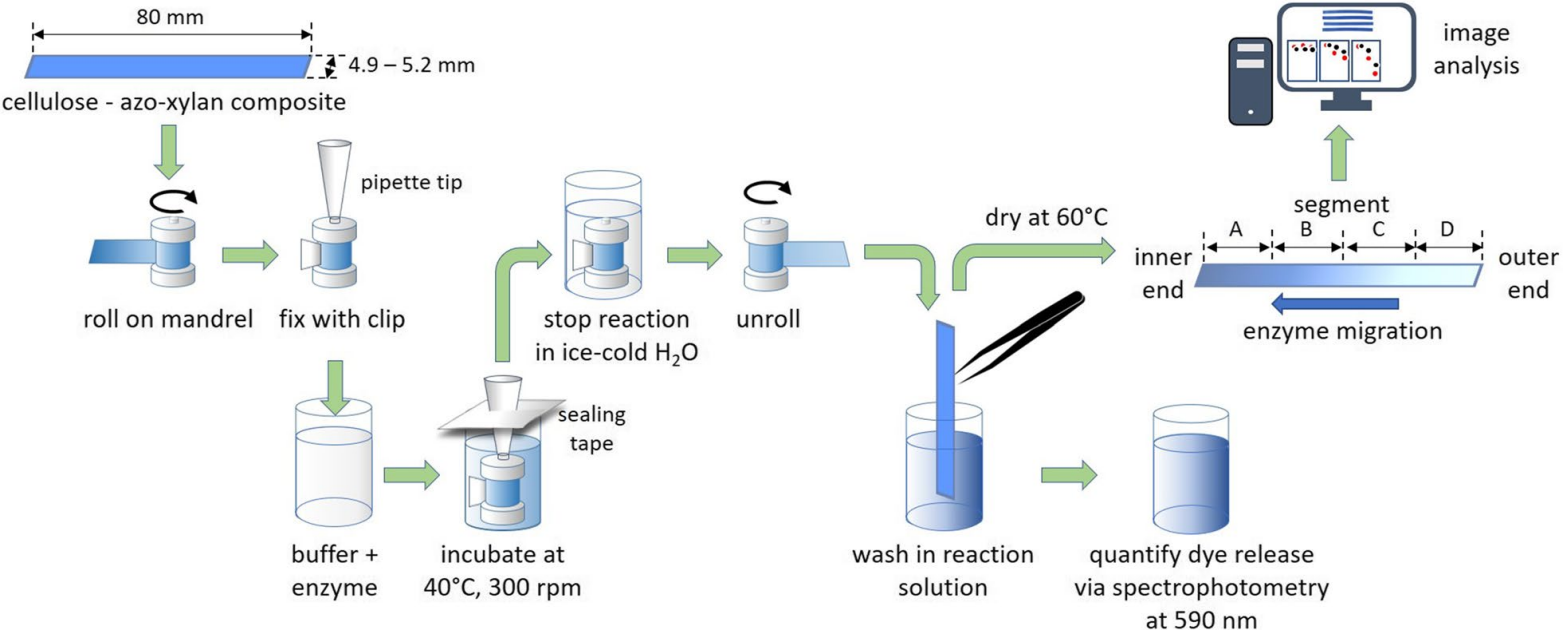
Schematic of the experimental set-up of the PACER assay. The range in composite width reflects the precision of the paper cutter and had no measurable impact on enzyme performance

Heteroxylans are a diverse group of branched xylans with a backbone of β-(1 → 4)-linked d-xylopyranosyl (Xyl*p*) residues that can be substituted with α-l-arabinofuranose (Ara*f*), 4-*O*-methyl-α-d-glucuronic acid (MeGlc*p*A), acetyl groups, *p*-coumaric acid and ferulic acid, depending on the botanical source [45]. After cellulose, xylans are the most abundant polysaccharide found in lignocellulosic biomass; accordingly, for proof of concept of the PACER assay, we used azo-coupled xylan from birchwood for the impregnation of filter paper (Fig. 1). To initially evaluate azo-xylan retention on the cellulose filter paper, azo-xylan solutions were prepared at between 2.5 and 10% w/v in either milli-Q water or 0.5 M NaOH, and the absorbance of remaining azo-xylan in solution was measured spectrophotometrically at 590 nm. Best retention was observed after solubilizing azo-xylan in 0.5 M NaOH and retention did not increase at azo-xylan concentrations above 5% w/v. Accordingly, the cellulose/azo-xylan composites were prepared using a 5% azo-xylan (w/v) solution prepared in 0.5 M NaOH; acid hydrolysis of corresponding composites confirmed 7.2 ± 0.5% w/w xylan in the generated materials.

### Comparison of the accessibility and activity of three different xylanases

The PACER assay was used to compare three *endo*-β-1,4-xylanases differentiated by glycoside hydrolase family and occurrence of a family 2 carbohydrate-binding module type B (CBM2b). The enzymes were the glycoside hydrolase family 10 (GH10) xylanase from *Cellvibrio mixtus* (*Cm*Xyn10B; Megazyme), the GH11 xylanase from *Neocallimastix patriciarum* (*Np*Xyn11A; Megazyme), and the GH11 xylanase from *Thermobifida fusca* (*Tf*Xyn11A) [46] that comprises a C-terminal carbohydrate-binding module belonging to CBM family 2 (CBM2b) (http://www.cazy.org) (Table 1). Briefly, GH10 *endo*-xylanases bind two consecutive unsubstituted Xyl*p* residues and can generate terminally substituted xylo-oligosaccharides, whereas GH11 xylanases require three consecutive unsubstituted Xyl*p* residues and can generate internally substituted xylo-oligosaccharides [47, 48].

**Table 1:** Properties of enzymes used in this study

Enzyme	Organism	CAZY number	Molecular mass (kDa)	CBM	UNIPROT Accession number	Specific activity (U/mg)^*^
*Cm*Xyn10B	*Cellvibrio mixtus*	GH10	41.7	–	O68541	7.32
*Np*Xyn11A	*Neocallimastix patriciarum*	GH11	25.8	–	P29127	14.2
*Tf*Xyn11A	*Thermobifida fusca*	GH11	31.9	CBM2b	Q47QL8	8.06
*Pm25* (WT)	Unclassified; from termite gut metagenome	GH10	83.9	CBM4	S0DFK9	0.29
*Pm*25 Y213A Y378A (M5)			83.8	CBM4		0.29
*Pm*25∆CBMs (M6)			48.8	–		0.49
*Pm*25 E546A (M1)			83.9	CBM4		0.01

^*^Determined using azo-xylan at pH 5.0 and 40 °C

**Fig. 2:**
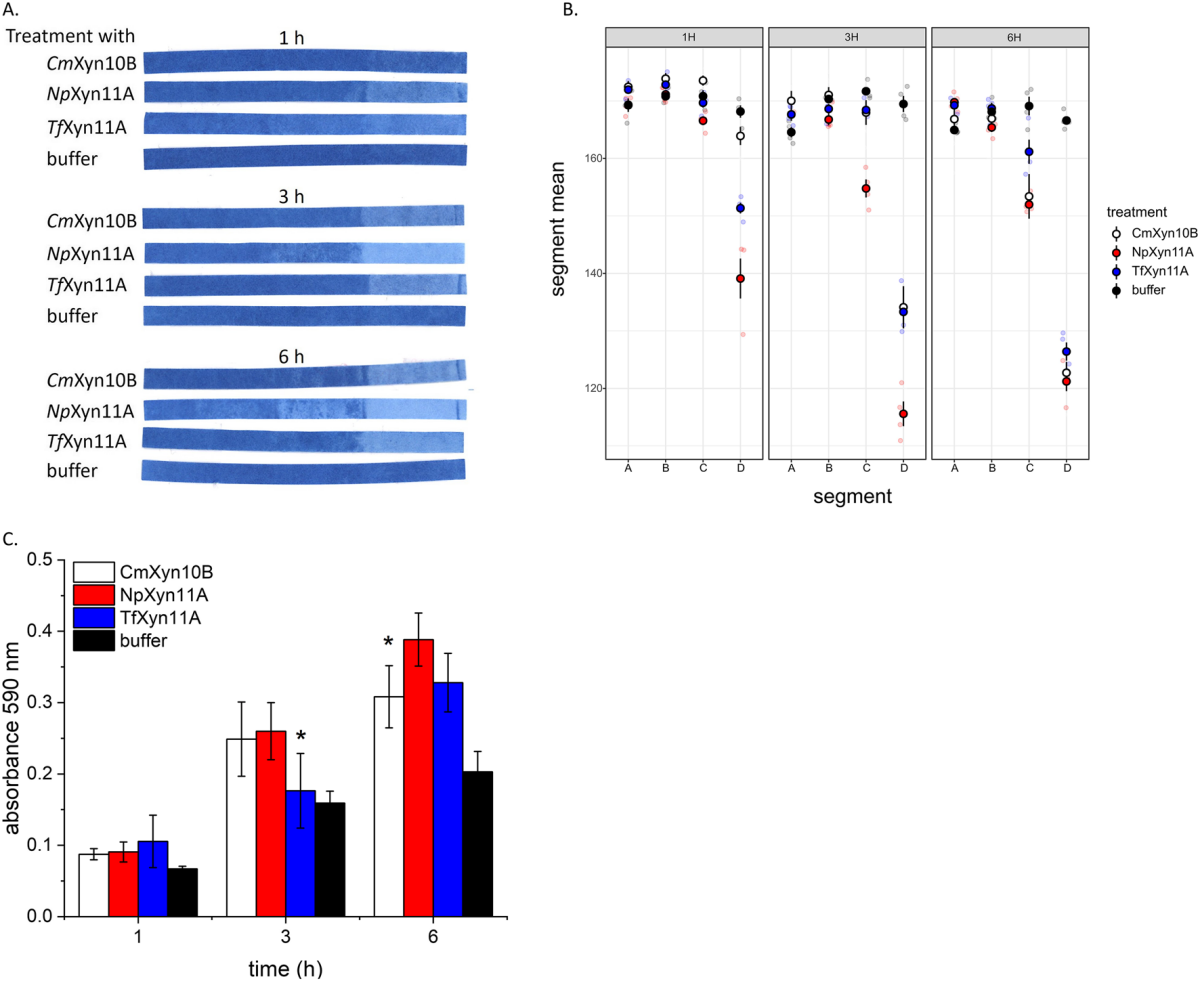
A comparison of three xylanases using the PACER assay. **A**–**C** Composites were incubated in 0.01 mg/ mL *Cm*Xyn10B, 0.005 mg/mL *Np*Xyn11A, 0.009 mg/mL *Tf*Xyn11A, or buffer only, at pH 5.0, 40 °C and 300 rpm for 1–6 h. **A** Representative cellulose/azo-xylan composites dried after treatment (Additional file 1: Fig. S5). **B** Average intensity values of dried cellulose/azo-xylan composites per segment. **C** Absorbance of the reaction solution at 590 nm recovered after composite incubation; statistically significant pairwise differences between *Cm*Xyn10B and *Np*Xyn11A, or *Tf*Xyn11A and *Np*Xyn11A are indicated by an asterisk (two-tailed t-test; *p* ≤ 0.05). For all experiments, *n* ≥ 4; errors bars correspond to standard deviation of mean

Following treatment with all three *endo*-β-1,4-xylanases, the cellulose/azo-xylan composite used in the PACER assay showed a discolouration that progressed over time from the right edge to the left edge of the strip (Fig. 2A, B). This was caused by enzymatic release of azo-coupled oligosaccharides and corresponds to enzyme migration from the outer layer (segment D) towards the inner layers (segment C-A) of the composite when rolled around the mandrel. Overall, *Np*Xyn11A migrated fastest and furthest through the cellulose/azo-xylan composite. The greater migration of *Np*Xyn11A compared to *Cm*Xyn10B and *Tf*Xyn11A was detected after each time point (Fig. 2A, B), even though *Cm*Xyn10B and *Tf*Xyn11A released similar levels of soluble product to *Np*Xyn11A after 1 h, and even after 3 h in the case of *Cm*Xyn10B (Fig. 2C).

This comparative analysis underscores the problem with equating soluble product release and enzyme accessibility to the corresponding substrate. Given that enzyme loadings used herein were based on units of activity, we reasoned that the different positions targeted by GH10 and GH11 would have negligible impact on enzyme migration through the constructed composites. Instead, the presence of a CBM, size of the catalytic domain, and number of Xyl*p* binding sites, likely impacted the diffusion of *Tf*Xyn11A, *Np*Xyn11A and *Cm*Xyn10B. *Tf*Xyn11A (32 kDa) contains a C-terminal CBM2b that binds both cellulose and insoluble xylan [46]. As a type B CBM, CBM2b of *Tf*Xyn11A is expected to primarily target the enzyme to the substrate (i.e., display a so-called targeting function) rather than promote fiber disruption [49, 50]. Accordingly, *Tf*Xyn11A migration through the cellulose/azo-xylan composite could be limited by dissociation from the substrate. Since neither *Cm*Xyn10B (42 kDa) or *Np*Xyn11A (26 kDa) comprise a CBM, the difference in their molecular weight likely drove the varying migration of these enzymes. It is also conceivable that the extended substrate binding surface formed by the higher number of Xyl*p* binding sub-sites expected in *Np*Xyn11A compared to *Cm*Xyn10B increased *Np*Xyn11A accessibility to the xylan substrate within the cellulose/azo-xylan composite. Future mutagenesis studies of *Np*Xyn11A, for example, could apply the PACER assay to directly evaluate the impact of an extended substrate binding site on substrate accessibility.

### Impact of loosenin-like proteins on *Np*Xyn11A migration

*Np*Xyn11A demonstrated greatest migration through the cellulose/azo-xylan composites and so was selected to investigate the potential of two loosenin-like proteins (PcaLOOL2 and PcaLOOL12 from *Phanerochaete carnosa*) for ability to boost enzyme accessibility to the xylan substrate [51]. Briefly, loosenins and loosenin-like proteins are microbial expansin-related proteins that comprise the *N*-terminal D1 domain characteristic of microbial and plant expansins [37, 52]. Similar to other microbial expansin-related proteins, loosenins reportedly weaken filter paper, disrupt cotton fibers, and boost cellulase action on lignocellulosic substrates by a non-lytic mechanism [37, 38].

**Fig. 3:**
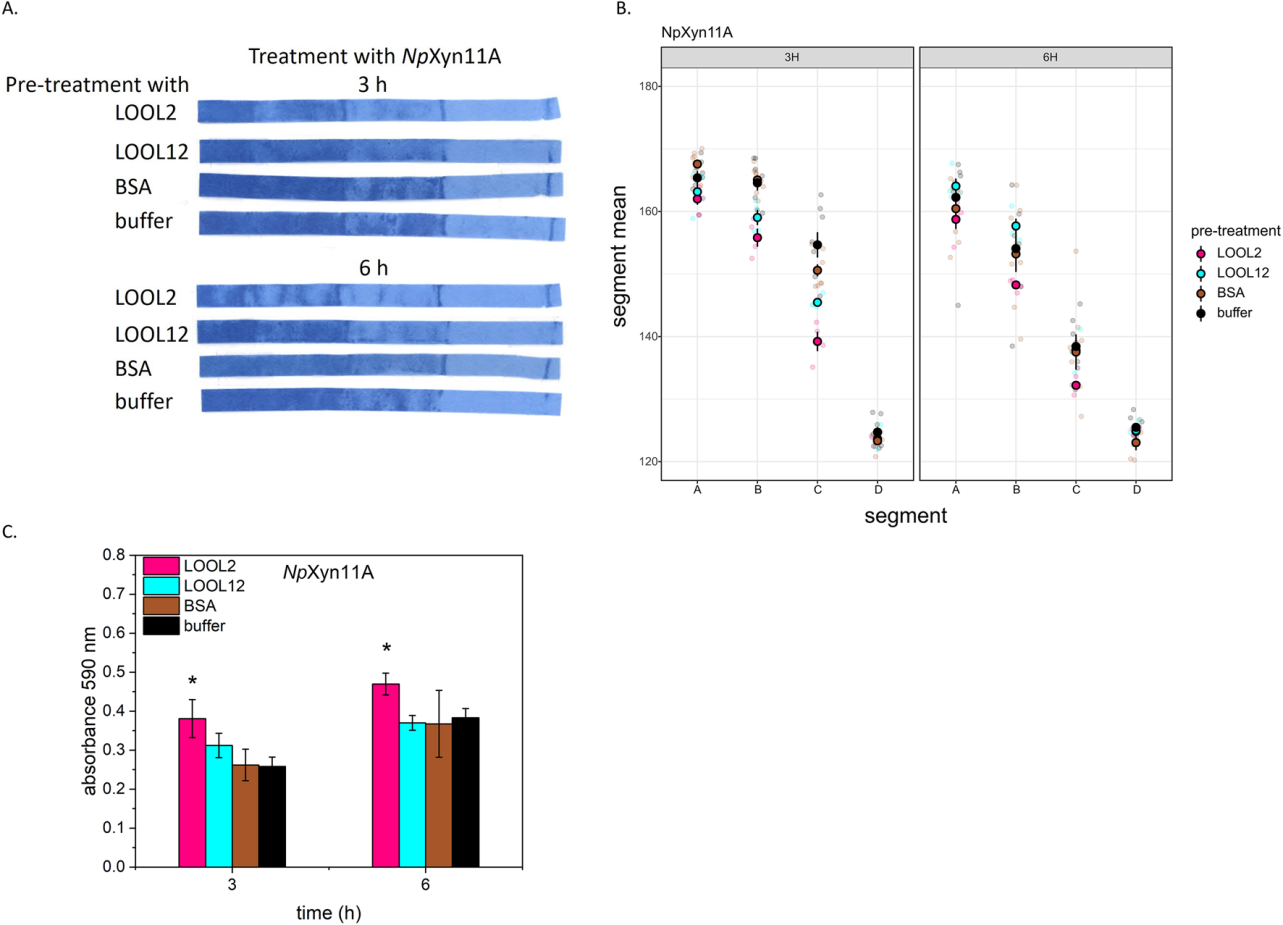
Impact of loosenin-like proteins on *Np*Xyn11A migration. Composites were pretreated with 0.1 mg/mL PcaLOOL2, PcaLOOL12, BSA or buffer only at pH 5.0 and room temperature for 24 h. This was followed by incubation in 0.005 mg/mL *Np*Xyn11A or buffer only, at pH 5.0, 40 °C and 300 rpm for 3–6 h. **A** Representative cellulose/azo-xylan composites dried after treatment. **B** Average intensity values of dried cellulose/azo-xylan composites per segment. **C** Absorbance of the reaction solution at 590 nm recovered after composite incubation; statistically significant differences compared to pretreatment with BSA are indicated by an asterisk (two-tailed t-test; *p* ≤ 0.05). For all experiments, *n* ≥ 4; error bars correspond to standard deviation of mean

Using the PACER assay, we observed the potential of PcaLOOL2 and PcaLOOL12 to increase *Np*Xyn11A migration through the constructed cellulose/azo-xylan composite. After 3 h of incubation, *Np*Xyn11A migration through the composites was more advanced in those pretreated with either PcaLOOL2 or PcaLOOL12 compared to composites pretreated with bovine serum albumin (BSA) (Fig. 3A, B). Moreover, the increased migration of *Np*Xyn11A through composites pretreated with PcaLOOL2 coincided with an increase in absorbance measurements of the resulting reaction solutions (Fig. 3C). Notably, the particular boosting effect of PcaLOOL2 on *Np*Xyn11A activity was not observed using azo-xylan in solution (Additional file 1: Fig. S1), and consistent with the predicted loosening effect of loosenin-like proteins, control reactions lacking *Np*Xyn11A showed low release of azo-xylan from composites treated with PcaLOOL2 and PcaLOOL12 alone (Additional file 1: Fig. S2).

### PACER for the characterization of a novel GH10 endo-β-1,4-xylanase and its mutants

Having demonstrated the potential of the PACER assay to uncover performance differences between well characterized xylanases, we then evaluated the potential of the assay to characterize a recently discovered multimodular xylanase. Briefly, the earlier metagenomic analysis of the fungus-growing termite, *Pseudacanthotermes militaris*, revealed a putative xylan utilization locus linked to the genus *Bacteroides* [53]. One of its open reading frames encodes a multimodular GH10, designated *P. militaris* 25 (*Pm*25). This enzyme is characterized by a discontinuous organization that includes the insertion of two tandem CBM4 domains (CBM4-1 and CBM4-2) within the catalytic GH10 xylanase domain (Table 1) [44].

To assess the impact of this unusual multidomain organization on enzyme migration, *Pm*25 and three constructs with modular deletions or point mutations were characterized using the PACER assay. Variant M1 (*Pm*25 E546A) harbors a point mutation in domain GH10b, reducing its activity on glucuronoxylan and arabinoxylan by two to three orders of magnitude [44]. Variant M5 (*Pm*25 Y213A + Y378A) carries two point mutations inactivating CBM4-1 and CBM4-2. Lastly, both CBM4 domains are deleted in M6 (*Pm*25∆CBMs) [44]. Given the particularly low activity of M1 on azo-xylan in solution (Table 1), the wild-type *Pm*25 and all variants were compared using the PACER assay on a molar basis.

**Fig. 4:**
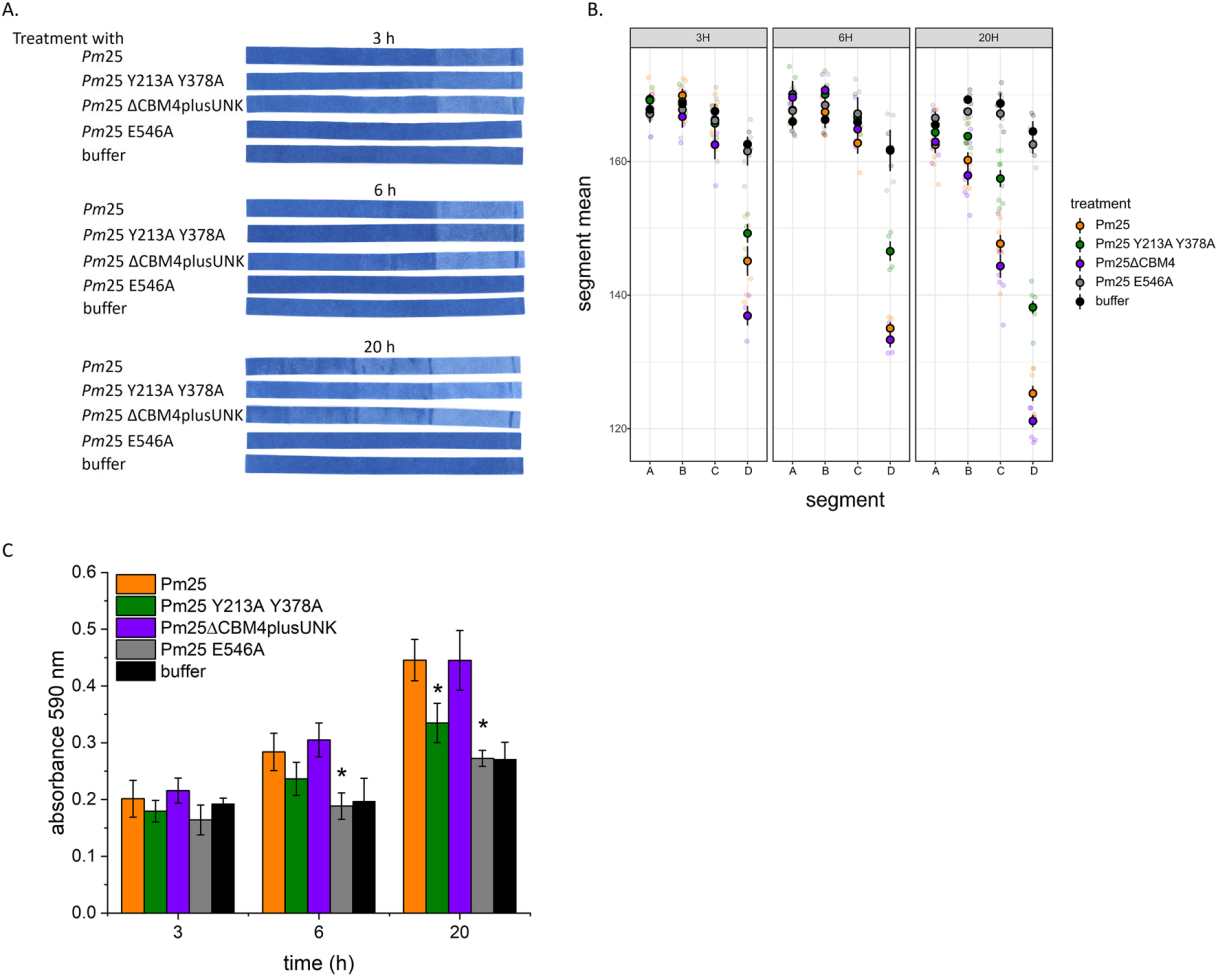
Characterization of the novel GH10 xylanase *Pm*25 and its mutants. Composites were incubated in 0.03 mg/ mL wild-type *Pm*25, 0.03 mg/mL variant M5 (*Pm*25 Y213A + Y378A), 0.017 mg/ mL variant M6 (*Pm*25∆CBMs), 0.03 mg/ mL variant M1 (*Pm*25 E546A), or buffer only, at pH 5.0, 40 °C and 300 rpm for 3–20 h. **A** Representative cellulose/azo-xylan composites dried after treatment. **B** Average intensity values of dried cellulose/azo-xylan composites per segment. **C** Absorbance of the reaction solution at 590 nm recovered after composite incubation; statistically significant differences compared to the wild-type *Pm*25 are indicated by an asterisk (two-tailed t-test; *p* ≤ 0.05). For all experiments, *n* ≥ 4; error bars correspond to standard deviation of mean

The low residual activity of the M1 variant was insufficient to promote detectable M1 migration through the cellulose/azo-xylan composite (Fig. 4A, B). More interesting, despite achieving similar levels of azo-xylan hydrolysis in solution (Additional file 1: Fig. S3), differences in wild-type *Pm*25, M5 and M6 performance after 3 h were clearly observed when using the PACER assay (Fig. 4A, B). The PACER assay also showed a clear correlation between activity and mobility, suggesting little to no passive diffusion of the enzyme through the system. The deletion of CBM4-1 and CBM4-2 leading to M6 significantly increased xylanase migration through the cellulose/azo-xylan composite, indicating that molecular weight outweighs the proximity effect gained by CBM4-1 and CBM4-2, at least at high substrate concentration [44]. Given the high apparent substrate concentration, the substantially lower migration of M5 compared to wild-type *Pm*25 was more surprising. Wu et al. [44] previously reported the lower activity of M5 compared to wild-type *Pm*25 on insoluble wheat bran; however, herein, differences in M5 and wild-type *Pm*25 migration were observed despite displaying similar levels of activity on azo-xylan (Fig. 4C). It is conceivable that active forms of CBM4-1 and/or CBM4-2 of *Pm*25 could impart both proximity and disruption effects to the wild-type *Pm*25 by extending the number of Xyl*p* binding sub-sites of the catalytic domain.

## Conclusions

The three-dimensional PACER assay was established to accelerate the discovery of non-catalytic proteins and protein domains that increase enzyme access to lignocellulosic substrates. Using a cellulose/azo-xylan composite to demonstrate the PACER concept, we could readily visualize the impact of xylan-binding modules and loosenin-like proteins on xylanase mobility and accessibility to the targeted substrate. The low cost, small scale, and option to create different composite materials, contribute to the advantages of the PACER assay. By using labeled proteins, composites could in future be constructed using defined and native lignocellulose components, and then visualized by tracking the labeled protein.

## Methods

### Substrates and chemicals

VWR® quantitative filter paper grade 454 (Cat.# 516-0857) was supplied by VWR (Radnor, PA, USA). Azo-xylan (birchwood powder; Cat.# S-AXBP) was obtained from Megazyme (Bray, Ireland). All other chemicals were reagent grade.

### Enzymes and non-catalytic proteins

*Cm*Xyn10B (recombinant *endo*-β-1,4-xylanase from *Cellvibrio mixtus*; Cat.# E-XYNBCM) and *Np*Xyn11A (recombinant *endo*-β-1,4-xylanase from *Neocallimastix patriciarum*; Cat.# E-XYLNP) were obtained from Megazyme. *Tf*Xyn11A (recombinant *endo*-β-1,4-xylanase from *Thermobifida fusca*) was kindly provided by T.V. Vuong [46]. All xylanases were purchased or recombinantly prepared as mono-component enzymes; protein purity was determined by SDS-PAGE (Additional file 1: Fig. S4). Bovine serum albumin (BSA) was obtained from Sigma-Aldrich (St. Louis, MO, USA; CAS# 9048-46-8).

PcaLOOL2 (loosenin-like protein 2 from *Phanerochaete carnosa*, GenBank code EKM55357.1) and PcaLOOL12 (loosenin-like protein 12 from *Phanerochaete carnosa*, GenBank code EKM51974.1) were recombinantly expressed in *Pichia pastoris* SMD1168H. Briefly, codon optimized genes encoding PcaLOOL2 or PcaLOOL12 were obtained as subcloned in pPICZαA plasmids with a C-terminal 6 × His tag (GenScript, Piscataway, NJ, USA). Transformants were grown in shake flasks in buffered glycerol-complex medium (BMGY; 100 mM potassium phosphate buffer (pH 6.0), 2% (w/v) peptone, 1% (w/v) yeast extract, 1.34% (w/v) yeast nitrogen base, 4 × 10^–5^% (w/v) biotin, 1% (v/v) glycerol) at 30 °C and 100 rpm until an OD_600_ of ~ 6 and then induced in buffered methanol-complex medium (BMMY; BMGY containing 0.5% (v/v) methanol instead of glycerol). Secreted recombinant proteins were purified from culture supernatants by FPLC using a GE Healthcare HisTrap™ FF Crude prepacked column (Thermo Fisher Scientific; Cat.# 11723219).

*Pm*25 (Xyn10C, *endo*-β-1,4-xylanase from the termite *Pseudacanthotermes militaris* gut metagenome, GenBank code CCO21036.1) and variants M6 (*Pm*25∆CBMs), M5 (*Pm*25 Y213A Y378A) and M1 (*Pm*25 E546A) were recombinantly expressed in *E. coli* Rosetta™ (DE3) pLysS as previously described [44].

### Enzyme activity determination

The activity of *end*o-β-1,4-xylanases on azo-xylan was determined according to the protocol provided by Megazyme with minor alterations. In brief, a 1% solution of azo-xylan was prepared in milli-Q H_2_O, 0.25 mL was transferred into a 15-mL Falcon™ conical centrifuge tube and pre-equilibrated in a ThermoMixer C set at 40 °C. Enzymes were diluted in 100 mM sodium acetate buffer (pH 5.0), 0.25 mL buffered enzyme preparation were added to the azo-xylan solution and thoroughly mixed. Tubes were incubated at 40 °C for 10 min, after which reactions were terminated by addition of 1.75 mL 96% (v/v) ethanol with vigorous stirring. Tubes were incubated at room temperature for 10 min and again thoroughly mixed. In the case of reaction blanks, ethanol was added prior to enzyme addition. Supernatants were recovered by centrifugation (3000 rpm for 10 min) and absorbance was analyzed spectrophotometrically at 590 nm. Activities were calculated with the Megazyme Mega-Calc™ using *Aspergillus niger endo*-β-xylanase as a reference.

### Preparation of cellulose/azo-xylan composites and confirmation of xylan content

Azo-xylan was dissolved in slightly heated 0.5 M NaOH to produce a 5% (w/v) solution and then stirred at room temperature for 1 h before transferring 6 mL of the solution to the bottom of a glass petri dish (Ø 190 mm). A pre-dried filter paper (VWR® quantitative filter paper grade 454; Ø 185 mm) was then submerged in the solution and covered with an additional 5 mL of the azo-xylan preparation. An even pressure was then applied by gently pressing the submerged filter paper with a second petri dish that had a slightly smaller diameter. After applying pressure to both sides of the filter paper, the paper was left to incubate in the azo-xylan solution for two more minutes before being placed between paper towels to remove excessive liquid. The resulting cellulose/azo-xylan composite was then dried for 10 min at 60 °C, washed in ~ 125 mL ice-cold 0.5 M acetic acid for 2 min, rinsed and washed in milli-Q H_2_O for 10 min, and then dried over night at 60 °C. The cellulose/azo-xylan composite was then cut into 4.9–5.2 mm × 80 mm strips using a guillotine paper trimmer. The weight percent of azo-xylan retained in the filter paper was verified by acid hydrolysis of cellulose/azo-xylan composites (*n* = 2). Briefly, for the acid hydrolysis, 10–11 mg of composite (two composites were analyzed in duplicate, *n* = 4) was prehydrolyzed with 72% H_2_SO_4_ (0.45 mL) at 30 °C for 1 h and then hydrolyzed with 1 M H_2_SO_4_ (10.9 mL) at 100 °C for 3 h. Samples were diluted 50 times and analyzed by high performance anion exchange chromatography (HPAEC) on a Dionex Ultimate 6000 system (Thermo Scientific, Sunnyvale, CA, USA) equipped with a CarboPac PA-1 column (2 mm × 250 mm ID) in combination with a CarboPac PA-1 guard column (2 mm × 50 mm ID) and PAD detector. Elution of monosaccharides (0.25 mL min^−1^) was performed with a multi-step-gradient using the following eluents: A: 0.1 M NaOH, B: 1 M NaOAc in 0.1 M NaOH, C: 0.2 M NaOH, and D: milli-Q water. Gradient was as follows: 0–20 min 16% A and 84% D; 20–25 min 45%A, 5%B, and 50%D; 15 min with 60%A and 40% B; 25–37 min 100% C (flow rate up to 0.35 mL min^−1^ over the first 2 min), and 37–49 min 16%A, 84%D (flow rate decreased to 0.25 mL min^−1^ over the first 2 min). The system was controlled using Chromeleon 7.2.9 software (Thermo Scientific, Sunnyvale, CA, USA).

### PACER assay

Cellulose/azo-xylan composites were soaked in 50 mM sodium acetate buffer (pH 5.0) for 3 min and then each strip was rolled tightly around a metal mandrel (Ø 6.0 mm, height 15.1 mm) before securing the outer end of the strip with a custom-made plastic clip [43]. The resulting mandrels were then placed in a 24-well round bottom microplate and fixed in an upright position using a pipette tip (Agilent Technologies, Santa Clara, CA, USA; Cat.# 202061-300) (Fig. 1).

To compare the migration of three different *endo*-β-1,4-xylanases, mandrels were incubated in a total reaction volume of 3 mL containing 0.01 mg/ mL *Cm*Xyn10B, 0.005 mg/ mL *Np*Xyn11A or 0.009 mg/ mL *Tf*Xyn11A in 50 mM sodium acetate buffer (pH 5.0). For all three enzymes, this corresponded to 71 mU/ mL. As a reference, mandrels were incubated in buffer only. Incubations were performed for 1—6 h on a ThermoMixer C set at 40 °C and 300 rpm.

The impact of loosenin-like proteins on the migration of *endo*-β-1,4-xylanases through cellulose/azo-xylan composites was investigated by submerging each strip (4.9–5.2 mm × 80 mm) in 0.5 mL of 50 mM sodium acetate buffer (pH 5.0) alone or containing 0.1 mg/ mL PcaLOOL2, PcaLOOL12, or BSA. After 24 h, each composite strip was rolled on a mandrel and incubated in 3 mL of 50 mM sodium acetate buffer (pH 5.0) alone or containing 0.005 mg/ mL (71 mU/ mL) *Np*Xyn11A. Incubations were performed for 0.5–6 h on a ThermoMixer C set at 40 °C and 300 rpm.

The PACER assay was also used to further characterize a novel GH10 *endo*-β-1,4-xylanase and its mutants. Here, mandrels were incubated in 3 mL of 50 mM sodium acetate buffer (pH 5.0) containing 0.03 mg/ mL wild-type *Pm*25, 0.03 mg/ mL of the M5 mutant (*Pm*25 Y213A Y378A), 0.017 mg/ mL of the M6 mutant (*Pm*25∆CBMs) or 0.03 mg/ mL of the M1 mutant (*Pm*25 E546A). As a reference, mandrels were incubated in buffer only. Incubations were performed for 0.5–20 h on a ThermoMixer C set at 40 °C and 300 rpm.

After each incubation, the mandrel was transferred to ice-cold milli-Q H_2_O for 3 min. Composites were then unrolled, washed three times in their corresponding reaction solution to release soluble products to the reaction supernatant, placed on a petri dish and dried over night at 60 °C. Absorbance of the reaction solution was analyzed spectrophotometrically at 590 nm.

### Image analysis

Dried composites were scanned at 600 × 600 dpi using Canon image RUNNER Advance C5250i system. The image processing was done with Fiji software [54, 55]. The scanned RGB images were converted to Hue Saturation Brightness (HSB) color space. The strips were segmented from the background pixels in the saturation channel using “Threshold tool” by choosing pixels with minimum intensity value of 80 and maximum value of 255. The saturation channel was chosen for the analysis because it contained most of the tone loss due to the enzymatic reaction in comparison to the other channels. From Fiji function “Analyze particles” the minimum particle size was set as 1000 pixels to remove wrongly selected pixels from the background. Fiji function “save XY coordinates” was used to extract the x and y coordinates of each pixel, as well as their intensity value in each strip. The data were stored as CSV files and imported to R studio [56] for further processing. X coordinates of the pixels were converted into percentages relative to full length of the strip and the split into four segments corresponding to turns of cellulose/azo-xylan composites around the mandrel. Mean intensity values of each segment with the standard error of the mean from at least four replicated strips per treatment were plotted. Processing, analysis and plotting of the data were done using Tidyverse 1.2.1, ggplot2 3.2.1, dplyr 0.8.3 R packages [56–59]. Code for extracting pixel information using Fiji and processing the data in R is deposited in GitHub and available through the link https://github.com/mipavici/PACERassay.

### Assay for in-solution hydrolysis of azo-xylan

Xylanase reactions using azo-xylan were optimized to ensure absorbance values were within the linear range of the assay. Final reactions contained 0.06% azo-xylan in 50 mM sodium acetate buffer (pH 5.0). For activity assays using *Np*Xyn11A, 0.001 mg/ mL *Np*Xyn11A were used and incubations were performed at 40 °C and 300 rpm for 10 min. *Np*Xyn11A activity on azo-xylan were also measured after pre-incubation of the azo-xylan solution with 0.003 mg/ mL PcaLOOL2, PcaLOOL12 or BSA in a ThermoMixer C set at 25 °C and 300 rpm for 24 h. For activity assays using *Pm*25 and corresponding variants, 0.03 mg/ mL *Pm*25, 0.03 mg/ mL *Pm*25 Y213A Y378A (aka M5), 0.017 mg/ mL *Pm*25∆CBMs (aka M6) or 0.03 mg/ mL *Pm*25 E546A (aka M1) were used and reactions were incubated in a ThermoMixer C set at 40 °C and 300 rpm for 3, 6 and 20 h.

In all cases, reactions were terminated using 1 mL 96% (v/v) ethanol with vigorous stirring. In the case of reaction blanks, ethanol was added prior to enzyme addition. Supernatants were recovered by centrifugation (15,000 rpm for 10 min) and absorbance was analyzed spectrophotometrically at 590 nm.

## Supplementary Information

**Additional file 1:** **Figure S1.** Impact of loosenin-like proteins on NpXyn11A hydrolysis of azo-xylan in solution. **Figure S2.** Impact of loosenin-like proteins on cellulose/azo-xylan composites. **Figure S3.** In-solution hydrolysis of azo-xylan by Pm25 and its mutants. **Figure S4.** SDS-PAGE analysis of CmXyn10B (lane 2), NpXyn11A (lane 3) and TfXyn11A (lane 4). **Figure S5.** Replicate cellulose/azo-xylan composites after treatment for 1-h with (A) NpXyn11A or (B) TfXyn11A.

## Data Availability

All data generated or analyzed during this study are reported in this manuscript and its Additional file 1.
