# Supplementary material for: PACER: a novel 3D plant cell wall model for the analysis of non-catalytic and enzymatic responses
Source: Biotechnol Biofuels Bioprod. 2022 Mar 16;15:30. doi: 10.1186/s13068-022-02128-8 (PMC8928621; doi:10.1186/s13068-022-02128-8)
Supplement: Supplementary file 1 — Additional file 1: Figure S1. Impact of loosenin-like proteins on NpXyn11A hydrolysis of azo-xylan in solution. Figure S2. Impact of loosenin-like proteins on cellulose/azo-xylan composites. Figure S3. In-solution hydrolysis of azo-xylan by Pm25 and its mutants. Figure S4. SDS-PAGE analysis of CmXyn10B (lane 2), NpXyn11A (lane 3) and TfXyn11A (lane 4). Figure S5. Replicate cellulose/azo-xylan composites after treatment for 1-h with (A) NpXyn11A or (B) TfXyn11A. [file 13068_2022_2128_MOESM1_ESM.pdf]

ADDITIONAL FILE 1

Mareike Monschein[a], Edita Jurak[a,b], Tanja Paasela[c],

Taru Koitto[a], Vera Lambauer[a], Mirko Pavicic[c], Thomas Enjalbert[d],

Claire Dumon[d] and Emma R. Master[a,e,*]

[a] Department of Bioproducts and Biosystems, Aalto University, Kemistintie 1, 02150 Espoo, Finland.

[b] Department of Bioproduct Engineering, University of Groningen, Nijenborgh 4, 9747 AG Groningen, The Netherlands.

[c] Department of Agricultural Sciences, Viikki Plant Science Centre, University of Helsinki, P.O. Box 27, 00014, Helsinki, Finland.

[d] Toulouse Biotechnology Institute (TBI), Université de Toulouse, CNRS, INRAE, INSA, 31077 Toulouse, France.

[e] Department of Chemical Engineering and Applied Chemistry, University of Toronto, 200 College Street, Toronto, Ontario, M5S 3E5, Canada.

*Corresponding author. phone number: +1416-946-7861

e-mail address: emma.master@aalto.fi

Additional File : Figure S1. Impact of loosenin-like proteins on *Np*Xyn11A hydrolysis of azo-xylan in solution. Reactions were optimized to ensure absorbance values were within the linear range of the assay. The azo-xylan solution was treated with 0.003 mg/ mL PcaLOOL2, PcaLOOL12, BSA or buffer only at pH 5.0, 25°C for 24 h, followed by addition of 0.001 mg/ mL *Np*Xyn11A, or buffer only, and incubation at 40°C, 300 rpm for 10 min. After precipitation of non-hydrolyzed substrate, absorbance of the reaction solution was recorded at 590 nm. n=3, errors correspond to standard deviation of mean.

Additional File : Figure S2. Impact of loosenin-like proteins on cellulose/azo-xylan composites. Composites were treated with 0.1 mg/ mL PcaLOOL2, PcaLOOL12, BSA or buffer only at pH 5.0 and room temperature for 24 h. This was followed by incubation of the resulting mandrels at pH 5.0, 40°C and 300 rpm for up to 6 h (A) Representative cellulose/azo-xylan composites dried after treatment. (B) Average intensity values of dried cellulose/azo-xylan composites per segment. (C) Absorbance of the reaction solution at 590 nm recovered after composite incubation. n≥4, errors correspond to standard deviation of mean.

Additional File : Figure S3. In-solution hydrolysis of azo-xylan by *Pm*25 and its mutants. The azo-xylan solution was treated with 0.03 mg/ mL wild-type *Pm*25, 0.03 mg/ mL M5 (*Pm*25 Y213A + Y378A), 0.017 mg/ mL M6 (*Pm*25ΔCBMs), 0.03 mg/ mL M1 (*Pm*25 E546A), or buffer only, at pH 5.0 and 40°C, 300 rpm for 3-20 h. After precipitation of non-hydrolysed substrate, absorbance of the reaction solution was recorded at 590 nm. n≥4, errors correspond to standard deviation of mean.

Additional File : Figure S4. SDS-PAGE analysis of *Cm*Xyn10B (lane 2), *Np*Xyn11A (lane 3) and *Tf*Xyn11A (lane 4). Lane 1 shows the molecular weight standard.

Additional File : Figure S5. Replicate cellulose/azo-xylan composites after treatment for 1-h with (A) NpXyn11A or (B) TfXyn11A

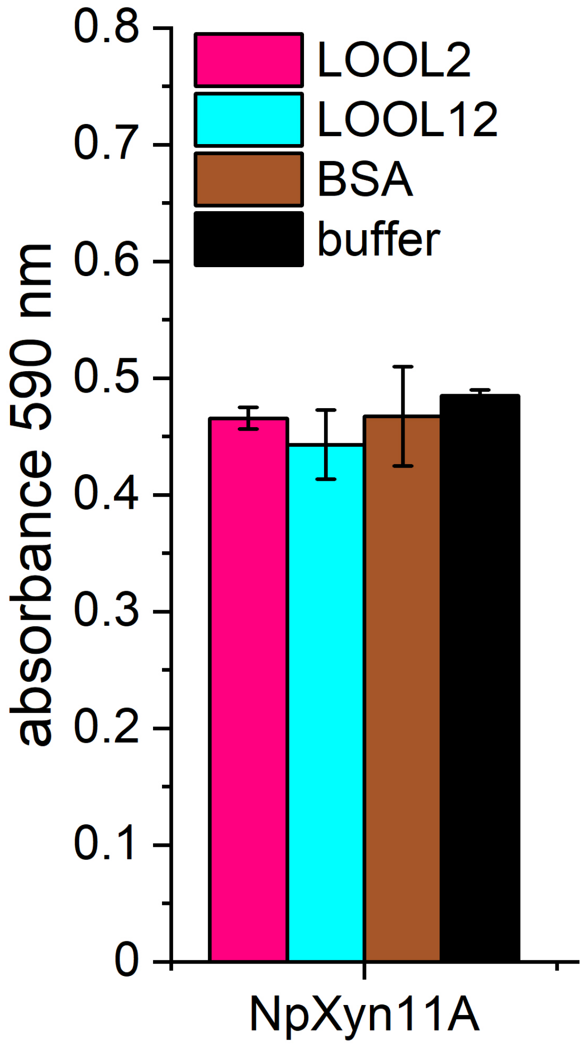
0.8
0.7
0.6
0.5
0.4
0.3
0.2
0.1
0
absorbance 590 nm
LOOL2
LOOL12
BSA
buffer
NpXyn11A

Figure S1

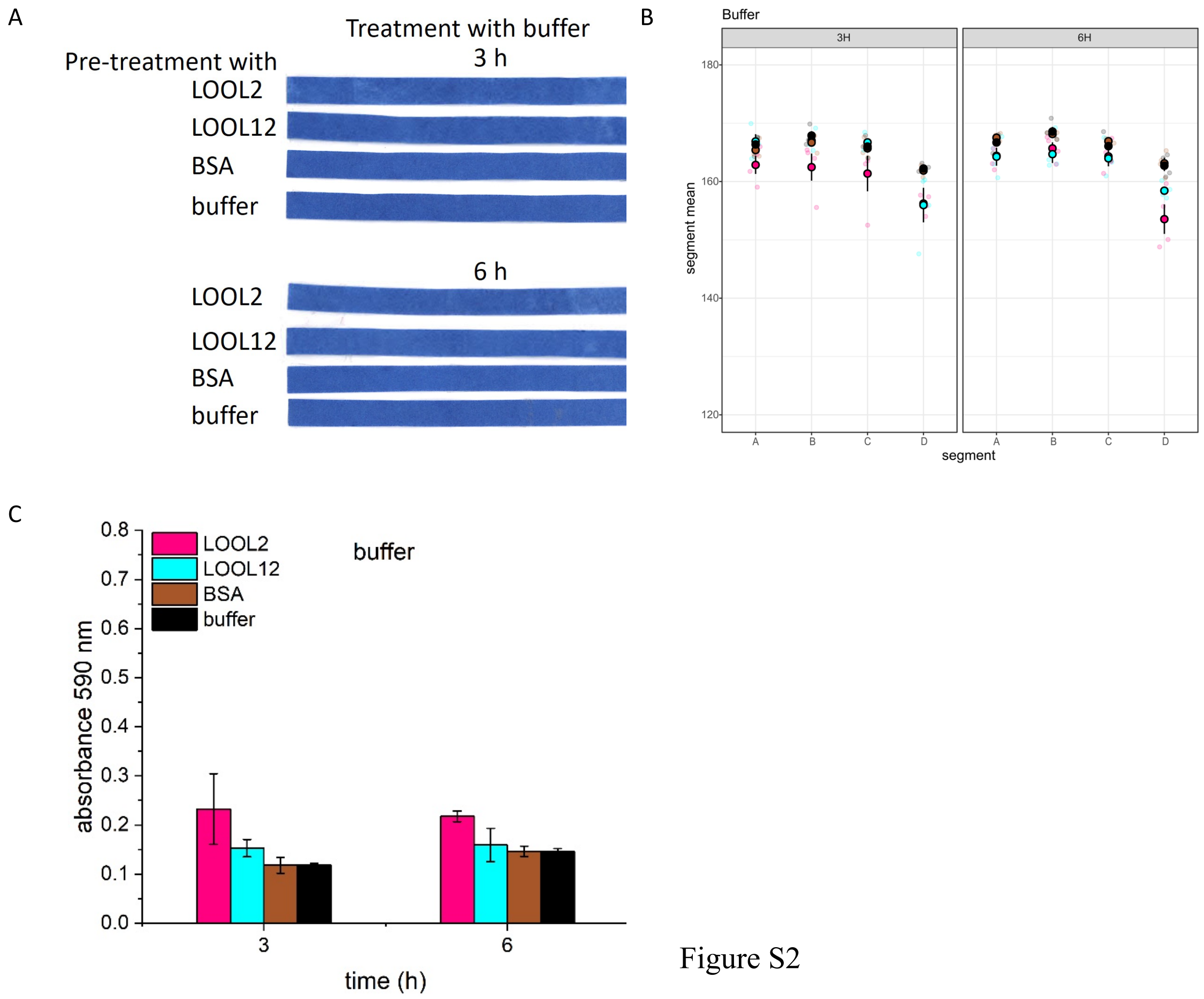

A
Treatment with buffer
Pre-treatment with
3 h
LOOL2
LOOL12
BSA
buffer
6 h
LOOL2
LOOL12
BSA
buffer
B
Buffer
3H
6H
segment mean
segment
C
absorbance 590 nm
LOOL2
LOOL12
BSA
buffer
buffer
time (h)

Figure S2

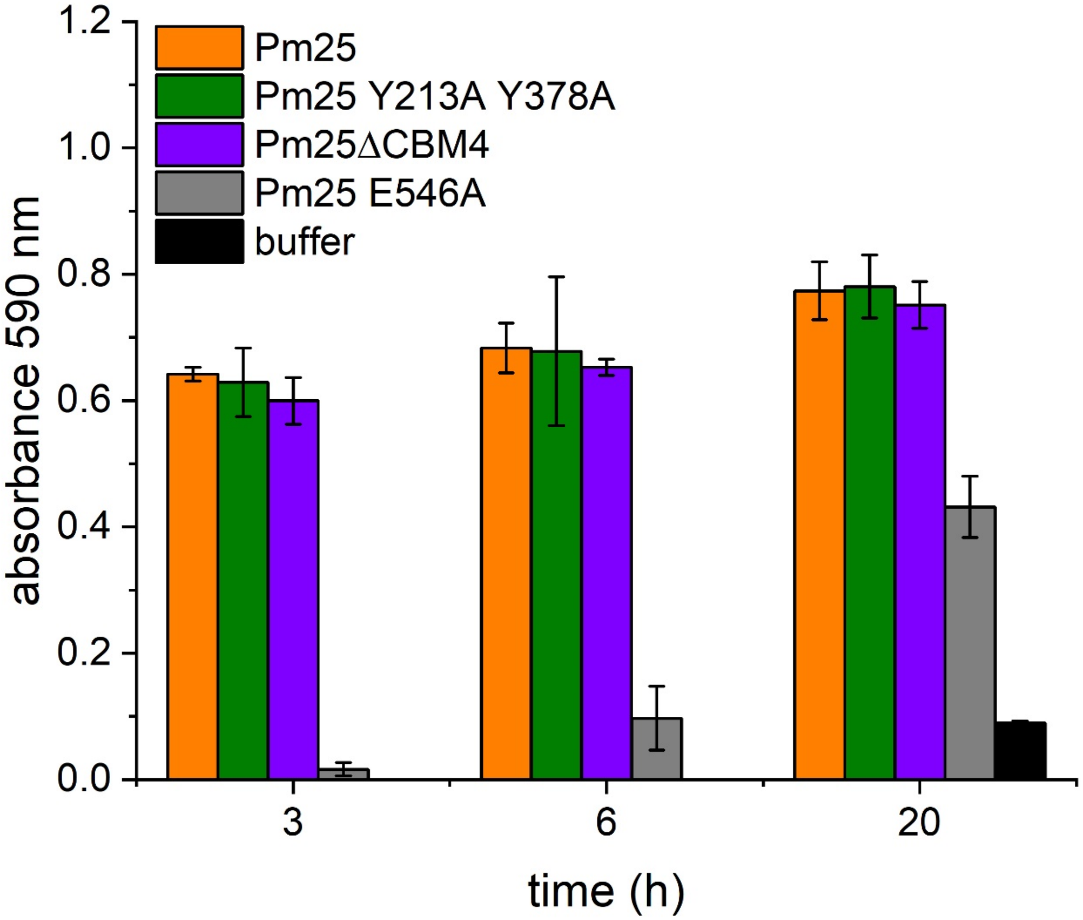
1.2
1.0
0.8
0.6
0.4
0.2
0.0
absorbance 590 nm
Pm25
Pm25 Y213A Y378A
Pm25ΔCBM4
Pm25 E546A
buffer
3
6
20
time (h)

Figure S3

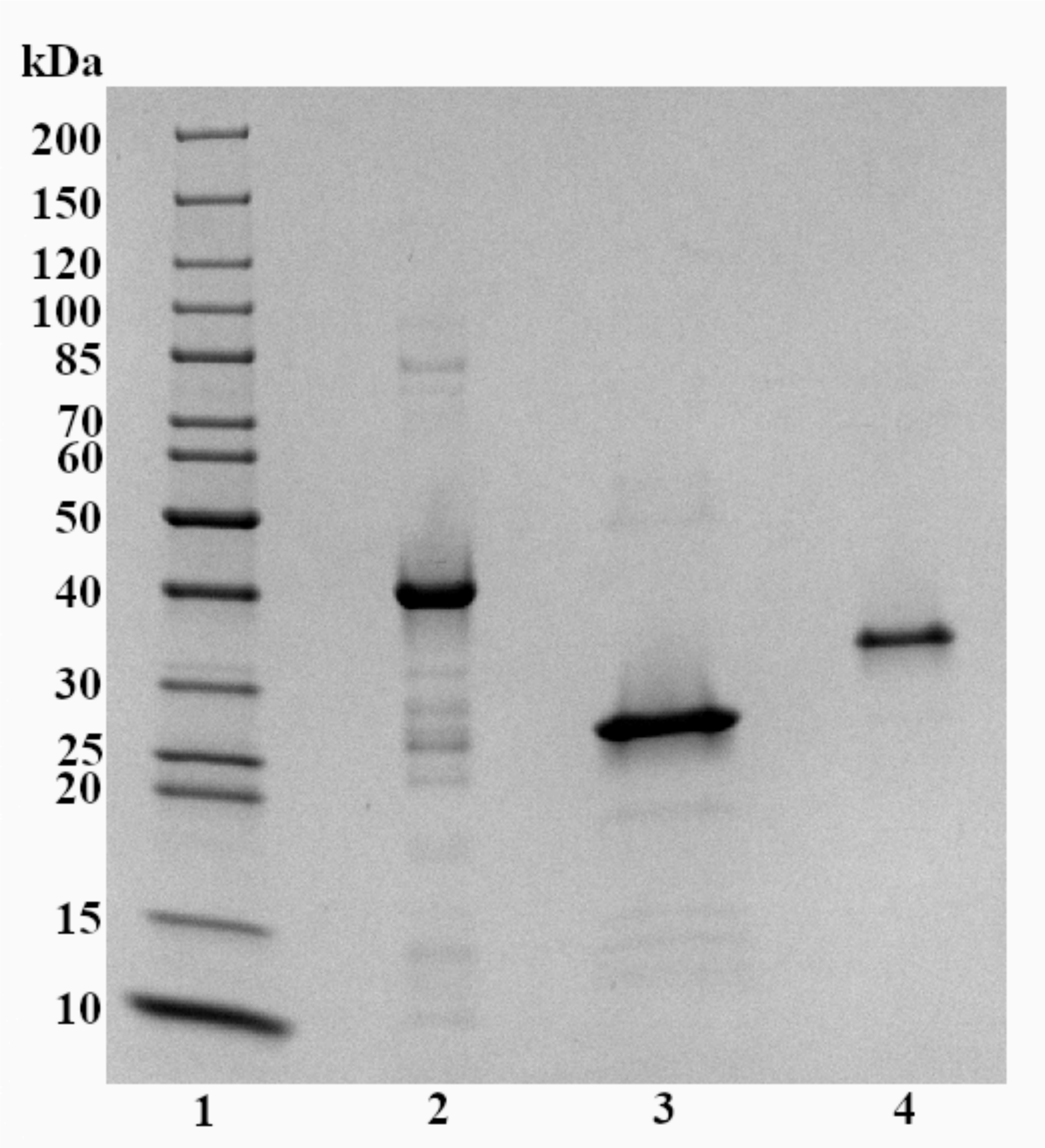

Figure S4

A. *Np*Xyn11A treatments

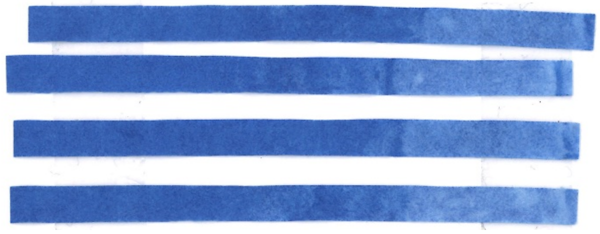

B. *Tf*Xyn11A treatments

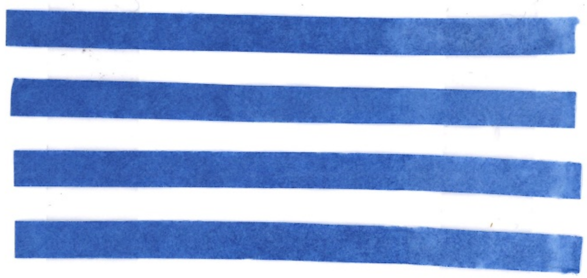

Figure S5
